# Extrahepatic Intraductal Papillary Neoplasms of Bile Duct and Klatskin Tumor: Simultaneous Occurrence and Recurrence?

**DOI:** 10.14309/crj.0000000000001522

**Published:** 2024-09-27

**Authors:** Mohammad Abuassi, Mikal Obed, Sebastian Dintner, Laszlo Füzesi, Mahmood Siyam, Anwar Jarrad, Aiman Obed

**Affiliations:** 1 Department of Internal Medicine, University of Central Florida, Gainesville, FL; 2 Department of Hepatology, Gastroenterology and Hepatobiliary/Transplant, Jordan Hospital, Ibn Sina University for Medical Sciences, Amman, Jordan; 3 Department of Pathology, Faculty of Medicine, University of Augsburg, Augsburg, Germany; 4 Department of General Surgery, Jordan Hospital, Ibn Sina University for Medical Sciences, Amman, Jordan

**Keywords:** cholangiocarcinoma, intraductal papillary neoplasm, Klatskin tumor, bile duct neoplasms, recurrence

## Abstract

This case report presents a 64-year-old woman with unique occurrence and recurrence of 2 different neoplastic entities, extrahepatic intraductal papillary neoplasm of bile duct and Klatskin tumor (hilar cholangiocarcinoma or central bile duct carcinoma), found simultaneously in close proximity. To date, this coexistence and recurrence with long survival time has not been reported. The patient in this case is remarkable for both the combination of intraductal papillary neoplasm of bile duct and cholangiocarcinoma, confirmed with specimen from the first surgical intervention in 2005, and recurrence of both tumors after 15 years and the patient's extraordinary survival with histological proven liver cirrhosis Child-Pugh class A. This case highlights the possibility of simultaneous occurrence and late recurrence of different neoplasms in the bile duct system and calls attention to the need for consideration in atypical cases.

## INTRODUCTION

Cholangiocarcinoma (CC) and intraductal papillary neoplasm of the bile duct (IPNB) are rare and potentially malignant tumors that present significant diagnostic and therapeutic challenges.^1^ CC can be intrahepatic, hilar, or extrahepatic. The most common type is hilar, involving the common hepatic duct bifurcation, and is referred to as Klatskin tumor. Klatskin tumors are associated with complicated diagnostic procedures, and their anatomical location makes the surgical site less accessible, resulting in higher unresectable rates.^2,3^ On the other hand, IPNB is a preinvasive, intraductal growing tumor originating from the bile duct epithelium, consisting of papillary and villous structures, with potential for malignancy.^4,5^ IPNB is classified as a biliary tumor subtype, according to the World Health Organization (WHO).^6^

In this report, we present an exceptional case of a patient with 2 separate neoplastic entities, IPNB and Klatskin tumor, found in close proximity within the right bile duct and duct bifurcation, yet remarkable long-term survival of 19 years. To the best of our knowledge, this is the first reported case of the coexistence of these 2 distinct neoplasms with late recurrence. The case report highlights the need for careful evaluation and monitoring of patients with IPNB and the importance of considering the possibility of multiple neoplastic entities within the bile duct system.

## CASE REPORT

**Figure 1.**
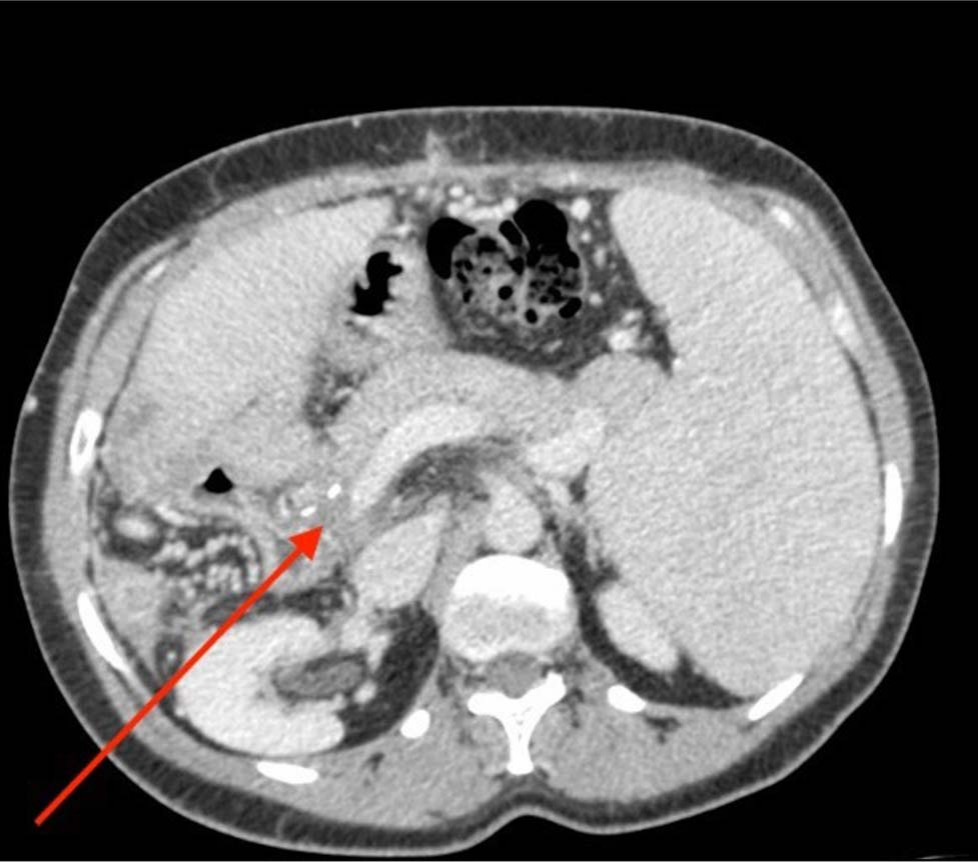
Postoperative computed tomography scan (2005) demonstrating portal vein thrombosis.

We report a case of a 64-year-old woman nonsmoker with history of hypertension, type 2 diabetes mellitus. The patient's surgical history includes splenectomy and metallic aortic valve replacement in 2011, as well as right hepatectomy with resection of the extrahepatic biliary system and left hepaticojejunostomy due to pathology proven combination of IPNB and intrahepatic CC in 2005, which was complicated postoperatively by portal vein thrombosis (Figure 1) that resolved after anticoagulation.

Initially, the follow-up course had been uneventful. But since 2017, regular insertion of a percutaneous transhepatic biliary drainage has been necessary every 3 months for recurrent cholangitis.

In early 2020, she was admitted via the emergency department due to severe fatigue, which occurred 1 month after the last percutaneous transhepatic biliary drainage. She reported experiencing nausea, nonbilious vomiting, and colicky abdominal pain in the right upper quadrant that was worsened with each meal. Biochemical testing revealed elevated inflammation parameters and liver enzymes, and her labs showed metabolic acidosis, hyponatremia, hyperkalemia associated with electrocardiographic changes (peaked T wave, flattened P wave, prolonged PR interval, and prolonged QRS duration), and acute kidney injury (Table 1). Overall findings were consistent with acute cholangitis, and percutaneous transhepatic cholangiography was placed to drain infection. Abdominal magnetic resonance cholangiopancreatography revealed moderate intrahepatic biliary dilation of the left hepatic duct with heterogeneous signal intensity and free contrast media flow in the biliary tree and jejunal loop, with no evidence of free peritoneal leakage. Based on these imaging findings, we suspected postsurgical fibrotic changes of the bile duct rather than recurrence of the Klatskin tumor.

**Table 1.** Laboratory results upon patient's admission on February 26, 2020

Test (abbreviation)	Patient	Normal range	Units
Hemoglobin (HB)	10.9	12–15.5 (female)	g/dL
White blood cell count (WBC)	9.41	4–11 × 10^3	cells/µL
Platelets (PLT)	460	150–400 × 10^3^	cells/µL
Bicarbonate (HCO3)	8.6	22–26	mmol/L
Chloride (Cl)	76	96–106	mmol/L
Phosphorus (P)	6.1	2.5–4.5	mg/dL
Creatinine (Cr)	1.75	0.7–1.2	mg/dL
Sodium (Na)	100	135–145	mmol/L
Urea (BUN)	98	7–20	mg/dL
Potassium (K)	7.03	3.5–5.0	mmol/L
Calcium (Ca)	10.74	8.5–10.5	mg/dL
Amylase	189	30–110	U/L
Lipase	400	13–60	U/L
Partial thromboplastin time (PTT)	44.4	25–35	Seconds
Prothrombin time (PT)	35.5	11–13.5	Seconds
International normalized ratio (INR)	2.84	0.8–1.2	—
Alanine aminotransferase (ALT)	34.7	7–56	U/L
Aspartate aminotransferase (AST)	52.3	10–40	U/L
Albumin	4.32	3.5–5.5	g/dL
Total protein (TP)	8.5	6.0–8.3	g/dL
Total bilirubin (TBIL)	1.09	0.3–1.2	mg/dL
Direct bilirubin (DBIL)	0.92	0–0.3	mg/dL
Gamma glutamyl transferase (GGT)	705	9–48	U/L
Alkaline phosphatase (ALP)	482	44–147	U/L
pH	7.2	7.35–7.45	—
Partial pressure of oxygen (PaO2)	125.2	75–100	mm Hg
Partial pressure of carbon dioxide (PaCO2)	22.6	35–45	mm Hg

**Figure 2.**
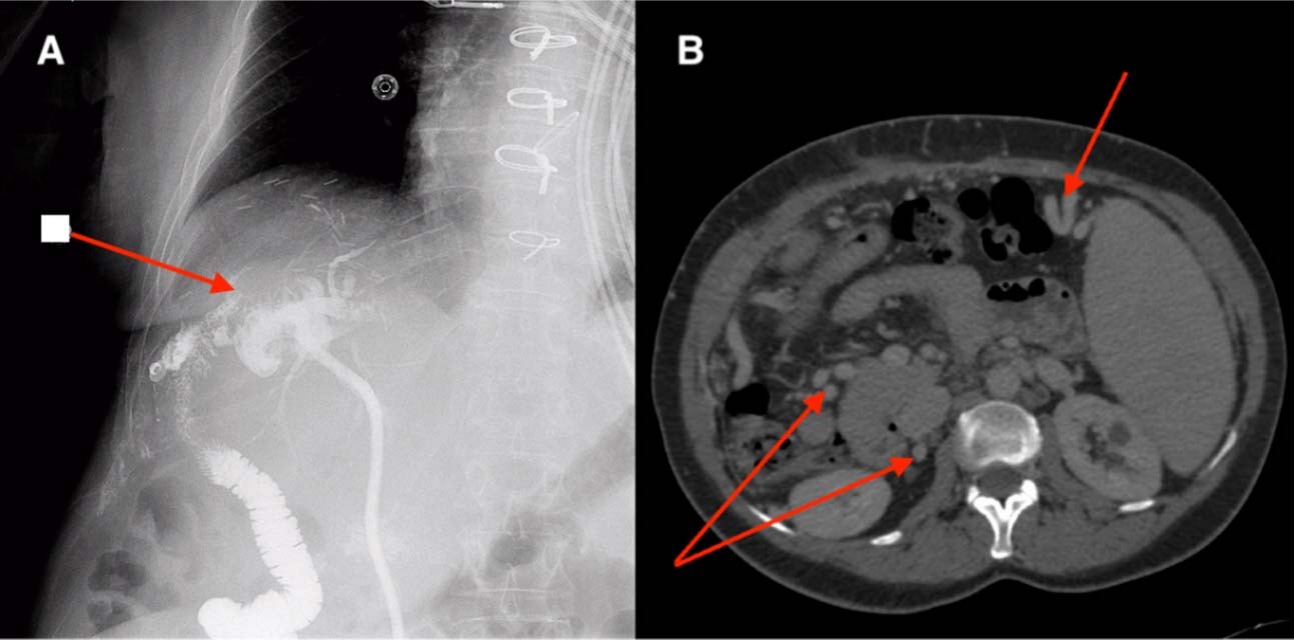
(A) Preoperative cholangiogram depicting filling defect and intrahepatic ducts; (B) computed tomography scan revealing splenomegaly and prominent venous collaterals.

**Figure 3.**
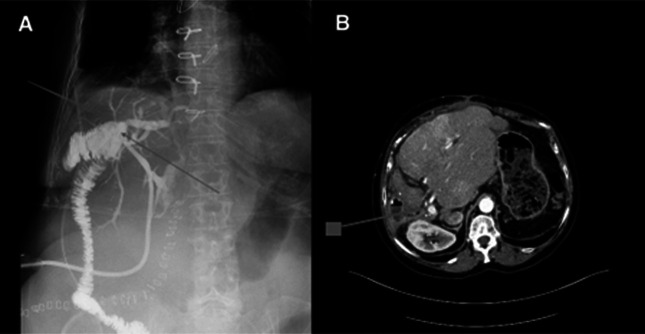
(A) Postoperative cholangiogram displaying absence of filling defect and intrahepatic ducts; (B) computed tomography scan illustrating patent portal vein and cirrhotic remnant in left liver lobe.

**Figure 4.**
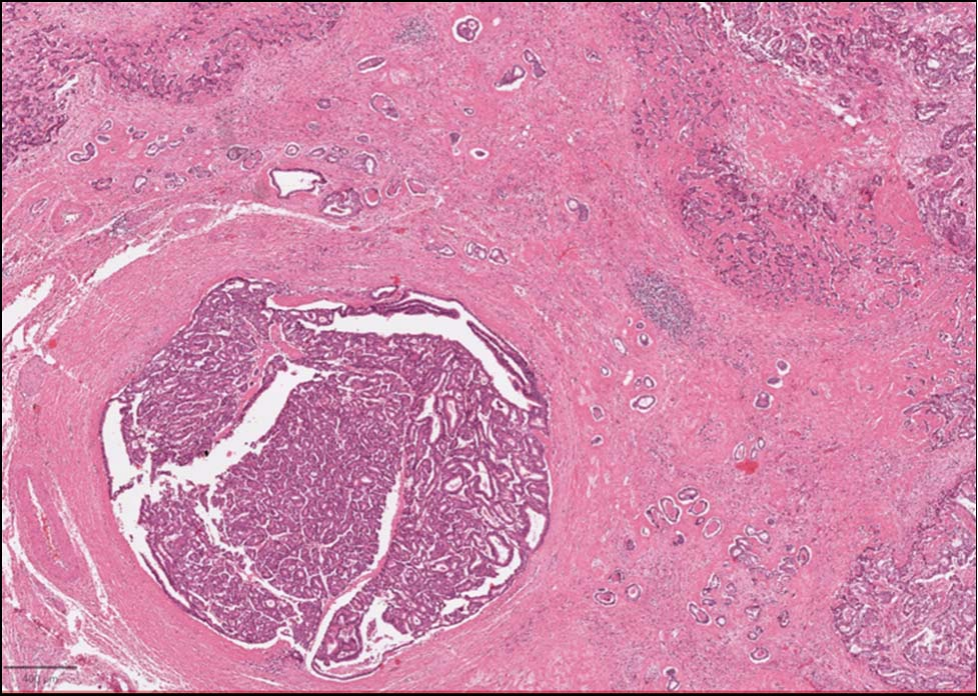
Co-occurrence of intraductal papillary neoplasm of the biliary tract (lower left section) and poorly differentiated cholangiocarcinoma (upper left section and the upper and lower right sections).

One month later, we performed a redo hepaticojejunostomy. Intraoperatively, it took at least 4 hours to reach the anastomosis site due to severe adhesions. Collaterals around the cirrhotic liver were also observed, indicating the presence of portal hypertension. After complete mobilization of the hepaticojejunostomy, we observed intraluminal masses in the small bowel loop of the hepaticojejunostomy, which displayed a paved quality that could be lifted off the jejunal mucous membrane. A new hepaticojejunostomy was performed, and 50 cm of the small bowel was resected (Figures 2 and 3). Samples were sent for final histopathological examination and showed irregular and bulky, with a gleaming gray/whitish surface on macroscopic examination, confirming IPNB and intrahepatic CC recurrence (Figure 4). Papillary tumor cells of IPNB respected the biliary duct wall. On the other hand, perihilar CC (pCC) was poorly differentiated and showed a spatial distance to IPNB. The remaining liver showed no focal lesions, but a coarse surface quality suggested chronic liver disease. Further molecular analysis of the new and old samples from the patient was carried out after passing internal Next-Generation Sequencing testing quality control. Two pathogenic single nucleotide variants in the FGFR2 (NM_022970.3) gene and a copy number gain of the MYC transcription factor were identified in all 3 specimens (Table 2).

**Table 2.** Patient's tumor characteristics and genetic alterations

No	Location	Age at diagnosis	Mutation (variant allele frequency)	Copy number variation, copy number
1	Right bile duct	48	FGFR2, c.1147T>C, p.Cys383Arg (65)	MYC, 5
			FGFR2, c.1159T>G, p.Phe387Val (65)	
2	Left bile duct	64	FGFR2, c.1147T>C, p.Cys383Arg (34)	MYC, 5
			FGFR2, c.1159T>G, p.Phe387Val (34)	
3	Small intestine	64	FGFR2, c.1147T>C, p.Cys383Arg (57)	MYC, 5
			FGFR2, c.1159T>G, p.Phe387Val (56)	

Postoperative tests showed no signs of biliary obstruction, and the patient's recovery was uneventful. The patient has since shown up regularly for follow-up examinations and has been in good general condition.

## DISCUSSION

CC is a highly aggressive malignant tumor that commonly affects the biliary tract.^2^ It is often categorized as intrahepatic or extrahepatic, with the latter further divided into hilar/perihilar (Klatskin tumor) and distal subtypes. Among these, extrahepatic CCs are the most prevalent. A specific subtype of extrahepatic CC, pCC, is located in the extrahepatic biliary tree proximal to the cystic duct's origin. Due to its complicated diagnostic pathway and anatomical location, pCC is associated with higher rates of unrespectability.^7^ Notably, pCC accounts for approximately 50% of CC, followed by distal (40%), and intrahepatic ones (<10%).^8^

The etiology of CC is mainly linked to infections, including infections with the liver flukes *Clonorchis sinensis* (*C. sinensis*) and *Opisthorchis viverrini*.^9,10^ In addition, chronic infection with hepatitis B and C viruses (hepatitis B virus and hepatitis C virus) may also be associated with CC.^11,12^ In our patient, 2 pathogenic single nucleotide variants in the FGFR2 gene and a copy number gain of the MYC transcription factor were identified in all 3 specimens. This indicates that the cancer development in this patient is likely driven by these inherited or acquired genetic mutations, rather than the more commonly associated infectious causes such as liver fluke infections or chronic hepatitis B and C virus infections. Klatskin tumor is a rare type of tumor that arises from the bifurcation of the extrahepatic bile duct and is also known as hilar CC or central bile duct carcinoma (Klatskin tumor). It accounts for only 1 in 100,000 annual incidences and typically presents with fatigue, jaundice, and cachexia, indicating metastatic or advanced tumors.^13^ Most patients with Klatskin tumors present with biliary symptoms, primarily painless jaundice, and less frequently with cholangitis if no previous biliary intervention has been performed. However, cholangitis becomes more common after biliary intervention, as observed in our case during the patient's second presentation.^14^ Klatskin tumors are a subtype of extrahepatic CC and pose a diagnostic challenge due to the difficulties associated with their classification.^15,16^

IPNB is a rare, preinvasive, and potentially malignant tumor of the bile duct that presents significant diagnostic and therapeutic challenges. The latest edition of the WHO Classification of Digestive System Tumors (2019) defines IPNB as a tumor originating from the bile epithelium, consisting of papillary and villous structures.^17^ Before its inclusion in the 2010 WHO classification, various types of tumors were included in the IPNB group, such as papilloma/ductal papilloma, intraductal bile duct carcinomas, papillary carcinomas of the extrahepatic bile duct, cystadenomas, and biliary cystadenomas.^5^ Currently, IPNB includes only intraductal growing bile carcinomas and papillary carcinomas of the extrahepatic bile ducts that grow intraductally in the form of papillary structures.^18^ IPNB has been observed to recur in the bile duct after surgical resection. Despite negative testing of the initial resection stump of the bile duct, the recurrent intraductal papillary tumor presents with tissue image similar to the initial resection, resembling the characteristics of intraductal papillary mucinous neoplasm. In some cases, IPNB recurs after more than 10 years, appearing as new carcinoma, suggesting multicentric growth of intraductal papillary mucinous neoplasm.^19^ Therefore, a careful observation of the clinical course is necessary, with postoperative intraductal recurrence in mind. These findings highlight the need for continued surveillance of patients after surgical resection for IPNB to ensure early detection and prompt management of any recurrence.

The unique simultaneous occurrence and late recurrence in our case of 2 separate neoplastic entities, IPNB and Klatskin tumor, that were found in close proximity within the right bile duct and duct bifurcation have not been reported in literature before. The patient underwent right hepatectomy, bile duct resection, and hepaticojejunostomy with the left bile duct in 2005. In 2020, a surgical redo of the hepaticojejunostomy was required due to recurrent cholangitis and a narrowed anastomosis with an unclear cause.

In conclusion, this case underscores the effectiveness of surgical intervention in achieving optimal survival rates even in cases of simultaneous occurrence and late recurrence of distinct neoplasms within the bile duct system. It is of vital importance to report all cases of this nature to enable better characterization and optimization of patient outcomes.

## DISCLOSURES

Author contributions: M. Abuassi and M. Obed: Main Manuscript writing and data organization; M. Siyam: data collection from Jordan Hospital and Hadassah Medical Center; L. Fuzesi: reviewed and wrote the pathology section and slides; S. Dintner: performed and wrote the molecular and genetics sections of the paper; A. Jarrad and A. Obed: performed procedures and surgery, provided care and follow-up for the patient, final review of the manuscript. M. Abuassi is the article guarantor.

Financial disclosure: None to report.

Informed consent was obtained for this case report.
